# Analysis of the data on titration of native and peroxynitrite modified αA- and αB-crystallins by Cu^2+^-ions

**DOI:** 10.1016/j.dib.2020.105492

**Published:** 2020-04-19

**Authors:** Maryam Ghahramani, Reza Yousefi, Kazem Khoshaman, Sogand Sasan Moghadam, Boris Kurganov

**Affiliations:** a Protein Chemistry Laboratory (PCL), Department of Biology, College of Sciences, Shiraz University, Shiraz, Iran; b Bach Institute of Biochemistry, Research Center of Biotechnology of the Russian Academy of Sciences, 33, bld. 2 Leninsky Ave., Moscow 119071, Russia

**Keywords:** Human αA-crystallin, Human αB-crystallin, Peroxynitrite, Copper ion

## Abstract

The interaction of αA- and αB-crystallins with Cu^2+^ ion modulates their structure and chaperone-like activity which is important for lens transparency. Theoretical analysis of the dependences of fluorescence intensity of native αA- and αB-crystallins and αA- and αB-crystallins modified by peroxynitrite on concentration of Cu^2+^ ions has been carried out. It has been shown that one subunit of native αA-crystallin contains two equivalent Cu^2+^-binding sites. The microscopic dissociation constant for Cu^2+^–αA-crystallin complex (*K*_diss_) was found to be equal to 9.7 µM. For peroxynitrite modified αA-crystallin the *K*_diss_ value is equal to 17 µM. One subunit of native αB-crystallin contains two non-equivalent Cu^2+^-binding sites. The corresponding microscopic dissociation constants for Cu^2+^–αB-crystallin complexes (*K*_1_ and *K*_2_) were found to be equal to 0.94 and 36 µM. For peroxynitrite modified αB-crystallin the *K*_1_ and *K*_2_ values are equal to 4.3 and 70 µM, respectively.

Specifications table

**Table:** 

Subject	Biochemistry
Specific subject area	Human α-crystallin, Peroxynitrite, Fluorescence spectroscopy, Cu^2+^ ion
Type of data	Graphs of protein titration by Cu^2+^ -ion
How data were acquired	The Trp-fluorescence spectra of different α-crystallins with titration of increasing concentrations of Cu^2+^-ions were obtained by a Cary Eclipse fluorescence spectrophotometer.
Data format	Raw and analyzed
Parameters for data collection	The measurements of protein samples (0.15 mg mL^−1^) were done at 25 °C in buffer A and the protein samples were titrated with increasing concentrations of Cu^2+^-ions (0–300 µM).
Description of data collection	The Trp-fluorescence spectra of native and peroxynitrite modified proteins were measured between 300 and 500 nm with excitation at 295 nm using fluorescence spectrophotometer. The dependence of fluorescence intensity of different protein samples on the concentration of Cu^2+^-ions was assessed at 337 nm in the absence and presence of different concentrations of Cu^2+^.
Data source location	Shiraz University, Shiraz, Iran
Data accessibility	With the article as Supplementary data
Related research article	M. Ghahramani, R. Yousefi, K. Khoshaman, S.S Moghadama, B.I. Kurganov, Evaluation of structure, chaperone-like activity and protective ability of peroxynitrite modified human α-сrystallin subunits against copper-mediated ascorbic acid oxidation, Int. J. Biol. Macromol. 87 (2016) 208–221. https://doi.org/10.1016/j.ijbiomac.2016.02.040.

## Value of the data

• The affinity of native and peroxynitrite modified αA- and αB-crystallins to copper ions were characterized.
• Modification by peroxynitrite results in the decrease in the affinity of these proteins to copper ions.
• The obtained data can be used for interpretation of the effect of copper ions on chaperone-like activity of both native and peroxynitrite modified variants of these proteins.
• These data might be of beneficial to clinical researches particularly in the case of patients with diabetes mellitus and during aging which are accompanied with elevation of both copper ions and oxidative stress in the lenticular tissues.

## 1 Data

### 1.1 Analysis of data on titration of the protein by the specific ligand obtained by fluorescence method. Theory

Consider the approaches to the analysis of the data on titration of the protein (P) by the specific ligand (L) obtained by fluorescence method. It is assumed that the protein molecule contains *n* ligand-binding sites (Ω). The equilibrium $\Omega +L\overset{}{⇆}\Omega L$ is characterized by the microscopic dissociation constant:

(1) $$K_{\text{diss}}=\frac{\left[\Omega \right]\left[L\right]}{\left[\Omega L\right]}=\frac{\left({\left[\Omega \right]}_{0}-\left[\Omega L\right]\right)\left({\left[L\right]}_{0}-\left[\Omega L\right]\right)}{\left[\Omega L\right]},$$

where [Ω]_0_ and [Ω] are the total and equilibrium concentrations of ligand-binding sites, [L]_0_ and [L] are the total and equilibrium concentrations of the ligand. The fluorescence intensity (*I*) is composed of two terms, one of which is proportional to the free binding sites concentration and other is proportional to ΩL complex concentration [1]:

(2) I=α[Ω]+β[ΩL].

When [L] = 0, the initial value of fluorescence intensity is equal to *I*_0_: $I_{0}=\alpha {\left[\Omega \right]}_{0}$. When [L]_0_ → ∞, the limiting value of fluorescence intensity is equal to *I*_lim_: $I_{\lim}=\beta {\left[\Omega \right]}_{0}$. Thus, Eq. (2) can be written as follows:

(3) $$I=I_{0}\frac{\left[\Omega \right]}{{\left[\Omega \right]}_{0}}+I_{\lim}\frac{\left[\Omega L\right]}{{\left[\Omega \right]}_{0}}.$$

Let [P]_0_ be the initial molar concentration of the protein. The total molar concentration of ligand-binding sites [Ω]_0_ is equal to *n*[P]_0_. Taking into accounts Eqs. (1) and (3), we can obtain the following expression for fluorescence intensity as a function of total concentrations of the protein and ligand:

(4) $$I=I_{0}+\left(I_{0}-I_{\lim}\right)\frac{n{\left[P\right]}_{0}+{\left[L\right]}_{0}+K_{\text{diss}}-\sqrt{{\left(n{\left[P\right]}_{0}+{\left[L\right]}_{0}+K_{\text{diss}}\right)}^{2}-4n{\left[P\right]}_{0}{\left[L\right]}_{0}}}{2n{\left[P\right]}_{0}}.$$

Given the number of the ligand-binding sites in the protein molecule (*n*) this expression allows determining the microscopic dissociation constant *K*_diss_ from the titration data. If the *n* value is unknown, the following approach can be used to check the equivalence of the ligand-binding sites and estimate the value of *n*. Fluorescence measurements allows us to calculate the degree of saturation of the binding sites by the ligand (*Y*):

(5) $$Y=\frac{\left[\Omega L\right]}{{\left[\Omega \right]}_{0}}=\frac{1-I/I_{0}}{1-I_{\lim}/I_{0}}.$$

The expression for *K*_diss_ acquires the following form:

(6) $$K_{\text{diss}}=\frac{\left(1-Y\right)\left({\left[L\right]}_{0}-Yn{\left[P\right]}_{0}\right)}{Y}.$$

This expression can be transformed to the linear anamorphosis:

(7) $$\frac{{\left[L\right]}_{0}\left(1-Y\right)}{Y}=\left(n{\left[P\right]}_{0}+K_{\text{diss}}\right)-n{\left[P\right]}_{0}Y.$$

**Fig. 1:**
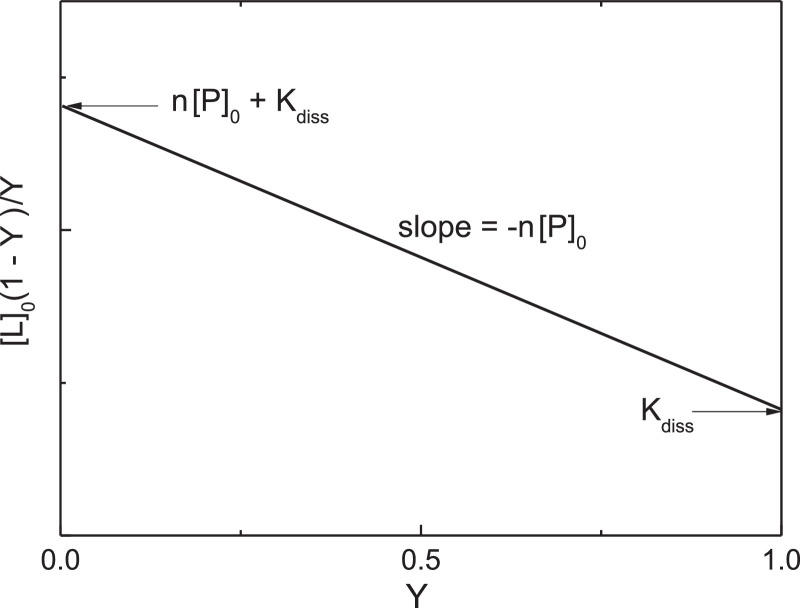
Analysis of data on titration of the protein by the specific ligand obtained by fluorescence method. Schematic representation of the [L]_0_(1 - *Y*)/*Y* versus *Y* plot for the case when the protein molecule contains *n* equivalent and non-interacting ligand-binding sites.

The [L]_0_(1 - *Y*)/*Y* versus *Y* plot is schematically represented in Fig. 1. The slope of the linear dependence is equal to –*n*[P]_0_. The length cut off on the ordinate axis is equal to (*n*[P]_0_ + *K*_diss_).

Consider the situation when the protein molecule contains two non-equivalent binding sites. The dependence of the degree of saturation *Y* on the equilibrium ligand concentration [L] has the following form:

(8) $$Y=\frac{\left[L\right]/K_{1}}{2\left(1+\left[L\right]/K_{1}\right)}+\frac{\left[L\right]/K_{2}}{2\left(1+\left[L\right]/K_{2}\right)},$$

where *K*_1_ and *K*_2_ are the dissociation constants for the complexes of the ligand with the corresponding binding sites. From this equation the [L] value can be expressed as a function of *Y*:

(9) $$\left[L\right]=\frac{-\left(K_{1}+K_{2}\right)\left(1-2Y\right)+\sqrt{{\left(K_{1}+K_{2}\right)}^{2}{\left(1-2Y\right)}^{2}+16K_{1}K_{2}Y\left(1-Y\right)}}{4\left(1-Y\right)}.$$

Taking into account that [L]_0_ = [L] + *Y*[Ω]_0_, we can obtain the following expression for [L]_0_(1 - *Y*)/*Y* as a function of *Y*:

(10) $$\frac{{\left[L\right]}_{0}\left(1-Y\right)}{Y}=\frac{-\left(K_{1}+K_{2}\right)\left(1-2Y\right)+\sqrt{{\left(K_{1}+K_{2}\right)}^{2}{\left(1-2Y\right)}^{2}+16K_{1}K_{2}Y\left(1-Y\right)}}{4Y}+\left(1-Y\right){\left[\Omega \right]}_{0}.$$

If it has been established that the protein molecule contains two non-equivalent ligand-binding sites, the determination of the dissociation constants *K*_1_ and *K*_2_ can be carried on using coordinates {[L]; *r*} where *r* is a number of the ligand molecules bound the protein molecule and [L] is the equilibrium ligand concentration $\left(\left[L\right]={\left[L\right]}_{0}-Y{\left[\Omega \right]}_{0}\right)$. The *Y* value is calculated from fluorescence data using Eq. (5). The dependence of *r* on [L] has the following form:

(11) $$r=\frac{\left[L\right]/K_{1}}{\left(1+\left[L\right]/K_{1}\right)}+\frac{\left[L\right]/K_{2}}{\left(1+\left[L\right]/K_{2}\right)}.$$

### 1.2 Titration of native αA-crystallin by Cu^2+^-ions

**Fig. 2:**
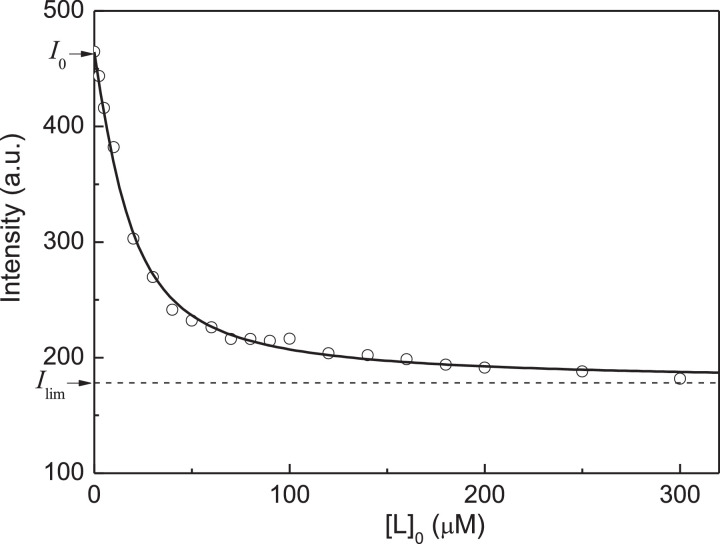
Titration of native αA-Cry by Cu^2+^-ions. Analysis of the titration data in coordinates {[L]_0_; Fluorescence intensity, *I*}. Points are the experimental points [2]. Solid curve was calculated from Eq. (4) at *I*_0_ = 464.5, *I*_lim_ = 178, [P]_0_ = 7.5 µM, *n* = 2 and *K*_diss_ = 9.7 µM.

Fig. 2 shows the dependence of fluorescence intensity of native αA-crystallin (αA-Cry) on the concentration of Cu^2+^-ions. The initial titration data are represented in Table S1 in supplementary materials.

**Fig. 3:**
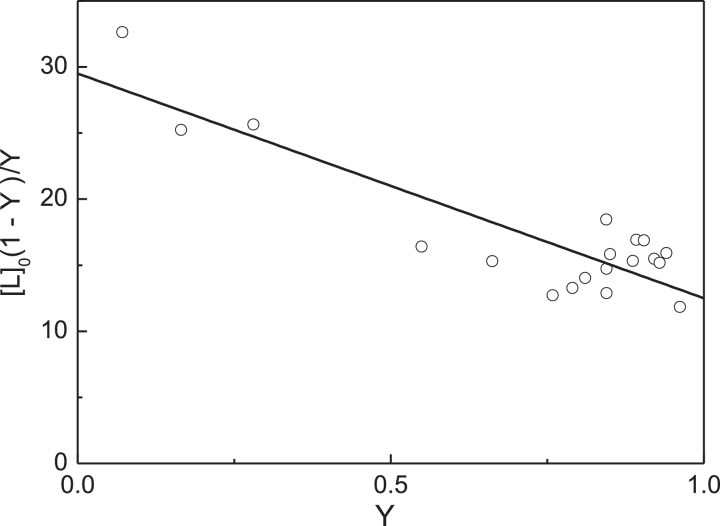
Titration of native αA-Cry by Cu^2+^-ions. Analysis of the titration data in coordinates {*Y*; [L]_0_(1 - *Y*)/*Y*}. Points are the experimental points. Solid line was calculated from Eq. (7) at *n*[P]_0_ = 17 µM and *K*_diss_ = 12.5 µM.

The primary analysis of the titration data can be carried out as follows. The limiting value of fluorescence intensity at [Cu^2+^]_0_ → ∞ was found by extrapolation to infinite ligand concentration in coordinates {1/[L]_0_; 1/(*I*_0_ – *I*)}: *I*_lim_ = 170.4 (*I*_lim_/*I*_0_ = 0.367). The initial concentration of native αA-Cry calculated on subunit, [P]_0_, in these experiments was 7.5 µM. The values of the degree of saturation of the protein by ligand at various concentrations of Cu^2+^-ions were calculated using Eq. (5). Fig. 3 shows the [L]_0_(1 - *Y*)/*Y* versus *Y* plot for these experimental data. The experimental points are represented in Table S2 in supplementary materials. The linear relationship between [L]_0_(1 - *Y*)/*Y* versus *Y* indicates that the ligand-binding sites in αA-Cry subunit are equivalent. The slope of the straight line passing through the experimental points was found to be -17 ± 2 µM (*R*^2^ = 0.7540). Comparing this value of the slope with [P]_0_ ([P]_0_ = 7.5 µM), one can conclude that *n* = 2.

Knowing the *n* value, we can analyze the dependence on fluorescence intensity on the ligand concentration using Eq. (4) without preliminary estimation of the *I*_lim_ value. The results of fitting Eq. (4) to the experimental data are shown in Fig. 2. The following values of parameters *I*_lim_ and *K*_diss_ were found: *I*_lim_ = 178 ± 2 and *K*_diss_ = 9.7 ± 0.7 µM (*R*^2^ = 0.9962).

### 1.3 Titration of peroxynitrite modified αA-Cry by Cu^2+^-ions

**Fig. 4:**
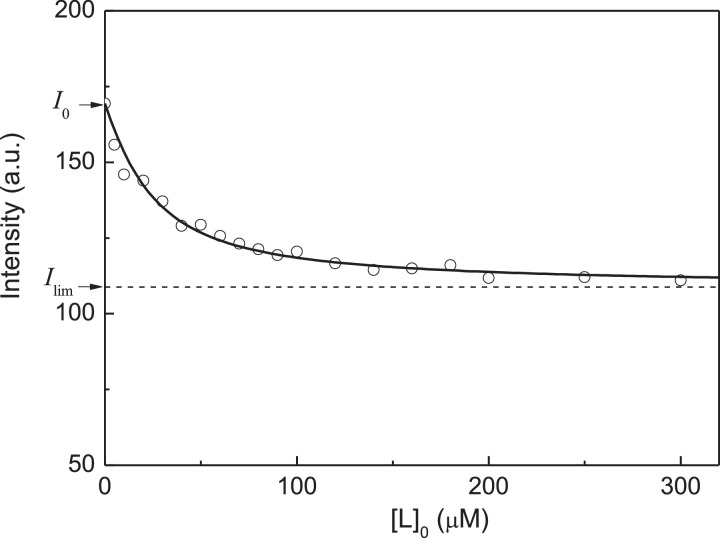
Titration of peroxynitrite modified αA-Cry by Cu^2+^-ions. Analysis of the titration data in coordinates {[L]_0_; Fluorescence intensity, *I*}. Points are the experimental points [2]. Solid curve was calculated from Eq. (4) at *I*_0_ = 169.4, *I*_lim_ = 108.8, [P]_0_ = 7.5 µM, *n* = 2 and *K*_diss_ = 17 µM.

Fig. 4 shows the dependence of fluorescence intensity of peroxynitrite modified αA-Cry on the concentration of Cu^2+^-ions. The initial titration data are represented in Table S3 in supplementary materials. The dependence of fluorescence intensity of peroxynitrite modified αA-Cry on the concentration of Cu^2+^-ions was analyzed with the assumption that αA-Cry subunit contains two equivalent ligand-binding sites. Eq. (4) was used for this purpose (Fig. 4). The initial concentration of peroxynitrite modified αA-Cry calculated on subunit, [P]_0_, in these experiments was 7.5 µM. The following values of parameters *I*_lim_ and *K*_diss_ were found: *I*_lim_ = 108.8 ± 1.5 and *K*_diss_ = 17 ± 2 µM (*R*^2^ = 0.9756).

### 1.4 Titration of native αB-Cry by Cu^2+^-ions

**Fig. 5:**
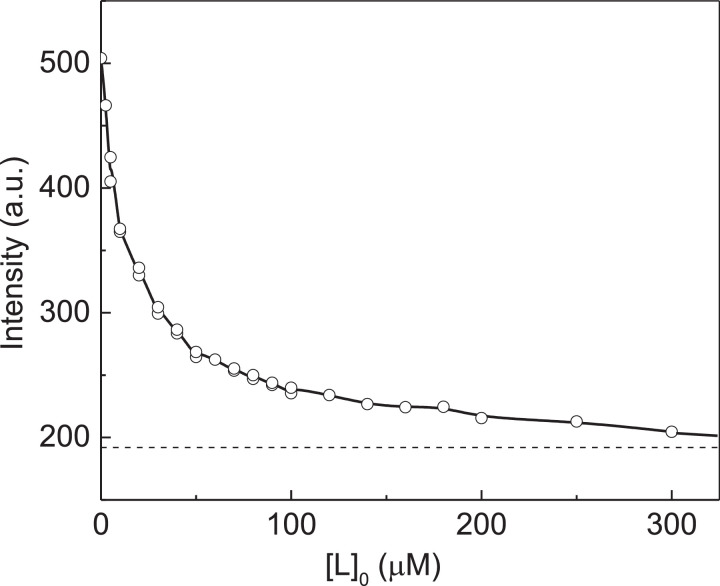
Titration of native αB-Cry by Cu^2+^-ions. The dependence of fluorescence intensity of native αB-Cry on the concentration of Cu^2+^-ions. Points are the experimental points [2]. Dashed line corresponds to the *I*_lim_ value.

Fig. 5 shows the dependence of fluorescence intensity of native αB-Cry on the concentration of Cu^2+^-ions. The initial titration data are represented in Table S4 in supplementary materials. The primary analysis of the dependence of fluorescence intensity of native αB-Cry on the concentration of Cu^2+^-ions was carried out as in the case of native αA-Cry. The limiting value of fluorescence intensity at [Cu^2+^]_0_ → ∞ was found by extrapolation to infinite ligand concentration in coordinates {1/[L]_0_; 1/(*I*_0_ – *I*)}: *I*_lim_ = 192.6 ± 1.9 (*I*_lim_/*I*_0_ = 0.382 ± 0.004).

**Fig. 6:**
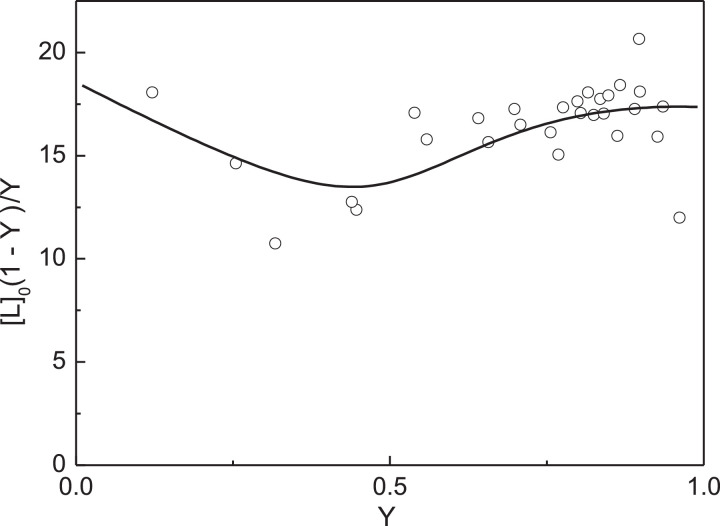
Titration of native αB-Cry by Cu^2+^-ions. Analysis of the titration data in coordinates {*Y*; [L]_0_(1 - *Y*)/*Y*}. Points are the experimental points. Solid curve was calculated from Eq. (10) at [Ω]_0_ = 17 µM, *K*_1_ = 0.8 µM and *K*_2_ = 33.9 µM.

The initial concentration of native αB-Cry calculated on subunit, [P]_0_, in these experiments was 7.5 µM. The values of the degree of saturation of the protein by ligand at various concentrations of Cu^2+^-ions were calculated using Eq. (5). Fig. 6 shows the [L]_0_(1 - *Y*)/*Y* versus *Y* plot for these experimental data. (The experimental points are represented in Table S5 in supplementary materials.).

**Fig. 7:**
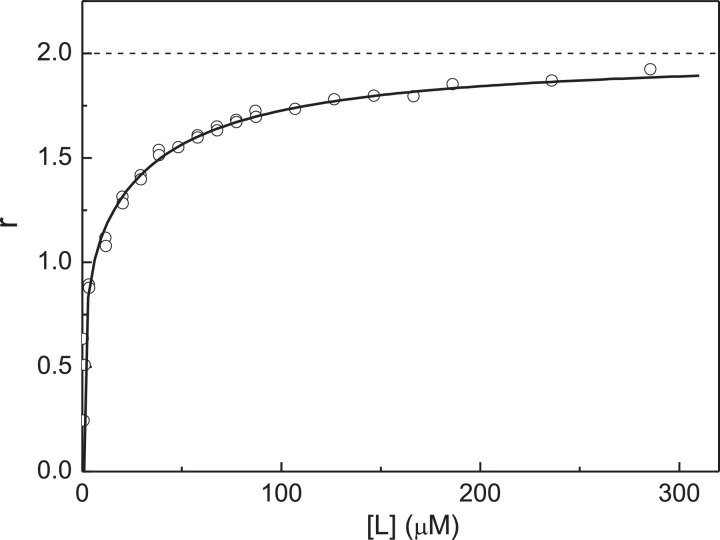
Titration of native αB-Cry by Cu^2+^-ions. Analysis of the titration data using the *r* versus [L] plot. Points are the experimental points. Solid curve was calculated from Eq. (11) at *K*_1_ = 0.94 µM and *K*_2_ = 36 µM.

The non-linear relationship between [L]_0_(1 - *Y*)/*Y* versus *Y* indicates that the ligand-binding sites in αB-Cry subunit are non-equivalent. As pointed out above, in the case of equivalent ligand-binding sites the length cut off on the ordinate axis is equal to ([Ω]_0_ + *K*_diss_). If *K*_diss_ << [Ω]_0_, this length is close to the [Ω]_0_ value. The fact that values of [L]_0_(1 - *Y*)/*Y* at low *Y* values on the plot represented in Fig. 6 are close to 15 µM allows us to assume the existence of two Cu^2+^-binding sites in αB-Cry subunit, the *K*_diss_ value for one of the ligand-binding sites is significantly less than the [Ω]_0_ value. Therefore, we described the dependence of [L]_0_(1 - *Y*)/*Y* on *Y* using Eq. (10). The value of [Ω]_0_ was found to be 17 ± 3 µM (*R*^2^ = 0.3132). Comparing this value of [Ω]_0_ with [P]_0_ ([P]_0_ = 7.5 µM), one can conclude that αB-Cry subunit actually contains two non-equivalent Cu^2+^-binding sites. Thus, to determine the values of the dissociation constants *K*_1_ and *K*_2_, the *r* versus [L] plot can be constructed (Fig. 7; the experimental points are represented in Table S6 in supplementary materials). When fitting Eq. (11) to the experimental dependence of *r* on [L], the following values of constants *K*_1_ and *K*_2_ were obtained: *K*_1_ = 0.94 ± 0.19 µM and *K*_2_ = 36 ± 3 µM (*R*^2^ = 0.9719).

### 1.5 Titration of peroxynitrite modified αB-Cry by Cu^2+^-ions

**Fig. 8:**
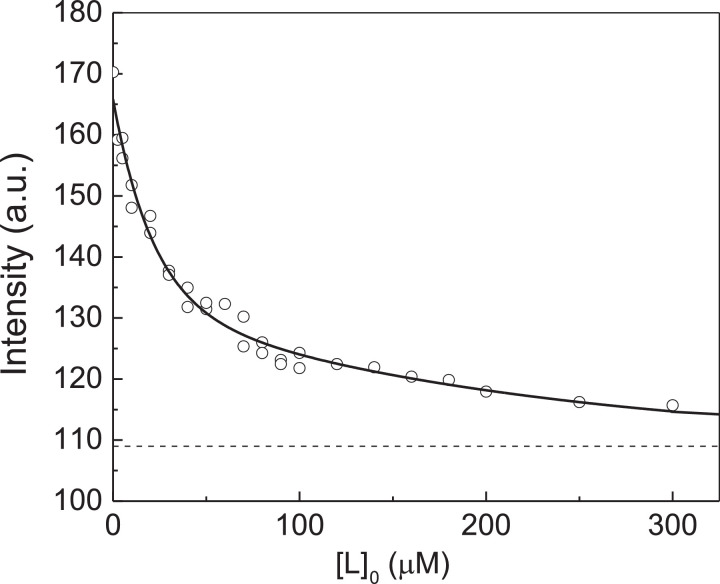
Titration of peroxynitrite modified αB-Cry by Cu^2+^-ions. The dependence of fluorescence intensity of peroxynitrite modified αB-Cry on the concentration of Cu^2+^-ions. Points are the experimental points [2]. Dashed line corresponds to the *I*_lim_ value.

Fig. 8 shows the dependence of fluorescence intensity of peroxynitrite modified αB-Cry on the concentration of Cu^2+^-ions. The initial titration data are represented in Table S7 in supplementary materials.

**Fig. 9:**
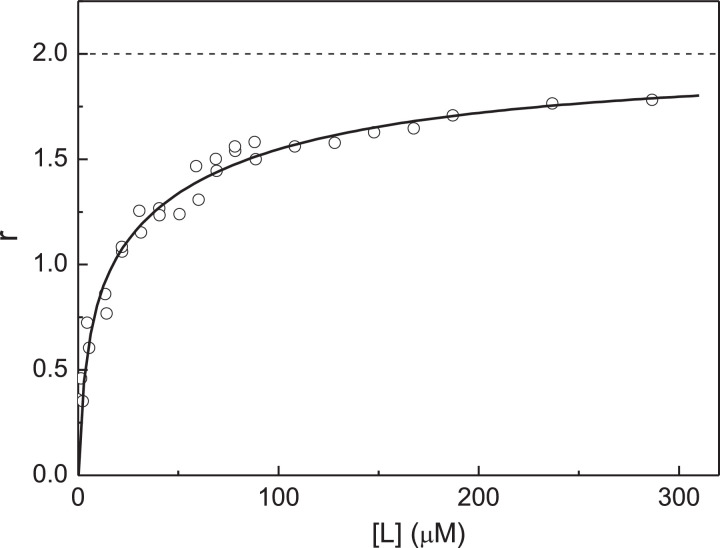
Titration of peroxynitrite modified αB-Cry by Cu^2+^-ions. Analysis of the titration data using the *r* versus [L] plot. Points are the experimental points. Solid curve was calculated from Eq. (11) at *K*_1_ = 4.3 µM and *K*_2_ = 70 µM.

The dependence of fluorescence intensity of peroxynitrite modified αB-Cry on the concentration of Cu^2+^-ions was analyzed with the assumption that αB-Cry subunit contains two non-equivalent ligand-binding sites. Eq. (11) was used for this purpose (Fig. 9; the experimental points are represented in Table S8 in supplementary materials). The initial concentration of peroxynitrite modified αB-Cry calculated on subunit, [P]_0_, in these experiments was 7.5 µM. The limiting value of fluorescence intensity at [Cu^2+^]_0_ → ∞, which is necessary to calculation of the *Y* and *r* values was determined by extrapolation to infinite ligand concentration in coordinates {1/[L]_0_; 1/(*I*_0_ – *I*)}: *I*_lim_ = 109 ± 1 (*I*_lim_/*I*_0_ = 0.64 ± 0.01). The following values of parameters *K*_1_ and *K*_2_ were found: *K*_1_ = 4.3 ± 0.9 µM and *K*_2_ = 70 ± 8 µM (*R*^2^ = 0.9592).

Thus, we use the [L]_0_(1 - *Y*)/*Y* versus *Y* plot only for selection of the binding model. Final calculations are carrying on in coordinates {[L]_0_; *I*} in the case of two equivalent binding sites (αA-Cry) or in coordinates {[L]; *r*} in the case of two non-equivalent binding sites (αB-Cry).

The results of fluorescence titration analysis have been used for characterization of the affinity of αA- and αB-crystallins and αA- and αB-crystallins modified by peroxynitrite to Cu^2+^ ions [2].

## 2 Experimental design, materials, and methods

### 2.1 Expression and purification of recombinant αA- and αB-Crys

The cDNA of human recombinant αA- and αB-Cry subunits which cloned into the bacterial vector pET-28b (+) was expressed in the BL21 (DE3) strain of *Escherichia coli* as described previously [3]. The centrifugation of cells which were harvested for 16 h after induction, was done at 5000 × *g* for 20 min at 4 °C. Then, the bacterial cell pellets were re-suspended in 25 mM Tris buffer, pH 7.2, containing 5 mM EDTA, 10 mM β-mercaptoethanol (β-ME), 100 mM NaCl and 0.01% NaN_3_ (lysis buffer). Then, the mixture was sonicated (five time for 30 s with 60% ultrasonic amplitude using a Bandelin Sonopuls sonicator, Berlin, Germany). The bacterial lysates were centrifuged at 8600 × *g* for 40 min at 4 °C and the supernatant dialyzed against 50 mM sodium phosphate buffer, pH 6.5. After that, the protein sample was loaded on a DEAE-cellulose (0.8 × 15 cm) anion exchange column which pre-equilibrated with the same buffer at 4 °C. The protein fractions were collected at a flow rate of 1 mL·min^−1^ in the presence of linear NaCl gradient 0.05–0.4 M in sodium phosphate buffer with a fraction size of 2 mL. The protein concentration was determined with Bradford assay and the highly purified fractions which assessed by SDS-PAGE (12% acrylamide) were collected and dialyzed overnight at 4 °C. The dialyzed sample (against 25 mM Tris buffer, pH 8.0, containing 0.5 mM EDTA, 10 mM β-ME and 0.01% NaN_3_) was then applied onto a Q-Sepharose (12.5 × 0.5 cm) anion exchange column which pre-equilibrated with the same buffer at 4 °C. The flow rate and fraction size of this column were fixed similar to the DEAE-cellulose column. The bound proteins were eluted with a 0–0.5 M NaCl gradient. The protein fractions were pooled and dialyzed against 25 mM Tris buffer, pH 8.0, containing 0.1 M NaCl, 0.5 mM EDTA, 10 mM β-ME and 0.01% NaN_3_. The concentrated protein samples were then applied onto a Sephacryl S-300HR gel filtration column (1.5 × 100 cm) that pre-equilibrated with the same buffer (4 °C, flow rate 0.25 mL min^−1^, fraction size 2 mL) [4], [5], [6]. The purity of the recombinant αA- and αB-Crys were confirmed with SDS-PAGE (12% gel). At the end, the highly purified protein fractions were collected and dialyzed against double distilled water (ddH_2_O) and stored at −20 °C until further use.

### 2.2 Peroxynitrite modification of recombinant αA- and αB-Crys

Synthesis of peroxynitrite was done according to the earlier studies [7,8]. The αA- and αB-Crys (2 mg mL^−1^) were incubated in the absence and presence of 7 mM peroxynitrite at room temperature for 30 min. Finally, the incubated solutions were individually dialyzed against ddH_2_O to remove excess peroxynitrite by using dialysis tube (cutoff of 10,000 Da). This experiment was done in 50 mM sodium phosphate buffer, pH 7.4, containing 10 mM HCO_3_^−^.

### 2.3 The fluorescence measurement of native and peroxynitrite modified αA- and αB-Crys

The Trp-fluorescence spectra of native and peroxynitrite modified αA- and αB-Crys (0.15 mg mL^−1^) were obtained between 300 and 500 nm after excitation at 295 nm using a Cary Eclipse fluorescence spectrophotometer [3,9]. The measurements were performed at 25 °C in 50 mM sodium phosphate buffer, pH 7.2 (buffer A) and the protein samples were titrated with increasing concentrations of Cu^2+^ (0–300 µM). The slit bandwidths were fixed at 10 nm in both channels. The dependence of fluorescence intensity of different protein samples on the concentration of Cu^2+^-ions was evaluated at 337 nm in the absence and presence of different concentrations of Cu^2+^.

### 2.4 Data analysis

Origin Pro 8.0 SR0 software was used for the calculations. To characterize the degree of agreement between experimental data and calculated values, we used the coefficient of determination *R*^2^ (see [10]).

## Appendix Supplementary materials

Image, application 1

Image, application 2
