# Supplementary material for: Analysis of the data on titration of native and peroxynitrite modified αA- and αB-crystallins by Cu2+-ions
Source: Data Brief. 2020 Apr 19;30:105492. doi: 10.1016/j.dib.2020.105492 (PMC7210411; doi:10.1016/j.dib.2020.105492)
Supplement: Supplementary file 1 [file mmc1.docx]

**Supplementary data**

**Analysis of the data on titration of native and peroxynitrite modified αA- and αB-Cry by Cu^2+^-ions**

Maryam Ghahramani^1^, Reza Yousefi*^1^, Kazem Khoshaman^1^, Sogand Sasan Moghadama^1^, Boris Kurganov^2^

^1^Protein Chemistry Laboratory (PCL), Department of Biology, College of Sciences, Shiraz University, Shiraz, Iran

^2^Bach Institute of Biochemistry, Research Center of Biotechnology of the Russian Academy of Sciences, 33, bld. 2 Leninsky Ave., Moscow 119071, Russia

* Corresponding author: Phone: +98 71 36137617, Fax: ++98 71 32280916.

E-mail: ryouseﬁ@shirazu.ac.ir

**Table S1. Titration of native αA-Cry by Cu^2+^-ions.** The dependence of fluorescence intensity of native αA-Cry on the concentration of Cu^2+^-ions.

| Cu^2+^ (μM) | Intensity at 337 nm (a.u.) |
| --- | --- |
| 0 | 464.5 |
| 2.5 | 443.6 |
| 5 | 415.9 |
| 10 | 382.0 |
| 20 | 303.0 |
| 30 | 269.8 |
| 40 | 241.4 |
| 50 | 232.1 |
| 60 | 226.2 |
| 70 | 216.2 |
| 80 | 216.2 |
| 90 | 214.5 |
| 100 | 216.3 |
| 120 | 203.8 |
| 140 | 202.2 |
| 160 | 198.5 |
| 180 | 193.8 |
| 200 | 191.2 |
| 250 | 188.1 |
| 300 | 181.6 |

**Table S2. Data on titration of native αA-Cry by Cu^2+^-ions represented in coordinates {*Y*; [L]_0_(1 ‑ *Y*)/*Y*}.** [L]_0_ is the total concentration of Cu^2+^-ions. *Y* is the degree of saturation of αA-Cry by Cu^2+^-ions**.**

| *Y* | [L]_0_(1 ‑ *Y*)/*Y* (μM) |
| --- | --- |
| 0.071 | 32.7 |
| 0.165 | 25.3 |
| 0.280 | 25.7 |
| 0.549 | 16.4 |
| 0.661 | 15.4 |
| 0.758 | 12.8 |
| 0.789 | 13.3 |
| 0.809 | 14.1 |
| 0.844 | 13.0 |
| 0.843 | 14.8 |
| 0.849 | 16.0 |
| 0.843 | 18.6 |
| 0.886 | 15.5 |
| 0.891 | 17.1 |
| 0.903 | 17.1 |
| 0.920 | 15.7 |
| 0.928 | 15.5 |
| 0.939 | 16.2 |
| 0.961 | 12.2 |

**Table S3. Titration of peroxynitrite modified αA-Cry by Cu^2+^-ions.** The dependence of fluorescence intensity of peroxynitrite modified αA-Cry on the concentration of Cu^2+^-ions.

| Cu^2+^ (μM) | Intensity at 337 nm (a.u.) |
| --- | --- |
| 0 | 169.4 |
| 2.5 | 166.4 |
| 5 | 155.8 |
| 10 | 145.9 |
| 20 | 143.9 |
| 30 | 137.1 |
| 40 | 129.0 |
| 50 | 129.4 |
| 60 | 125.7 |
| 70 | 123.1 |
| 80 | 121.3 |
| 90 | 119.3 |
| 100 | 120.4 |
| 120 | 116.6 |
| 140 | 114.4 |
| 160 | 115.0 |
| 180 | 116.0 |
| 200 | 111.8 |
| 250 | 112.1 |
| 300 | 111.0 |

**Table S4. Titration of native αB-Cry by Cu^2+^-ions.** The dependence of fluorescence intensity of native αB-Cry on the concentration of Cu^2+^-ions.

| Cu^2+^ (μM) | Intensity at 337 nm (a.u.) |
| --- | --- |
| 0 | 504 |
| 2.5 | 466 |
| 5 | 405 |
| 5 | 425 |
| 10 | 365 |
| 10 | 367 |
| 20 | 330 |
| 20 | 336 |
| 30 | 299 |
| 30 | 304 |
| 40 | 284 |
| 40 | 286 |
| 50 | 265 |
| 50 | 269 |
| 60 | 262 |
| 70 | 254 |
| 70 | 255 |
| 80 | 247 |
| 80 | 250 |
| 90 | 242 |
| 90 | 244 |
| 100 | 235 |
| 100 | 240 |
| 120 | 234 |
| 140 | 227 |
| 160 | 224 |
| 180 | 225 |
| 200 | 216 |
| 250 | 213 |
| 300 | 205 |

**Table S5. Data on titration of native αB-Cry by Cu^2+^-ions represented in coordinates {*Y*; [L]_0_(1 ‑ *Y*)/*Y*}.** [L]_0_ is the total concentration of Cu^2+^-ions. *Y* is the degree of saturation of αB-Cry by Cu^2+^-ions**.**

| *Y* | [L]_0_(1 ‑ *Y*)/*Y* (μM) |
| --- | --- |
| 0.122 | 18.06 |
| 0.318 | 10.75 |
| 0.255 | 14.62 |
| 0.447 | 12.37 |
| 0.439 | 12.76 |
| 0.559 | 15.79 |
| 0.539 | 17.08 |
| 0.657 | 15.65 |
| 0.641 | 16.81 |
| 0.708 | 16.50 |
| 0.699 | 17.26 |
| 0.769 | 15.04 |
| 0.756 | 16.13 |
| 0.776 | 17.34 |
| 0.804 | 17.07 |
| 0.799 | 17.63 |
| 0.825 | 16.97 |
| 0.816 | 18.06 |
| 0.841 | 17.04 |
| 0.835 | 17.75 |
| 0.862 | 15.95 |
| 0.848 | 17.92 |
| 0.867 | 18.41 |
| 0.890 | 17.26 |
| 0.898 | 18.10 |
| 0.897 | 20.66 |
| 0.926 | 15.91 |
| 0.935 | 17.38 |
| 0.962 | 11.99 |

**Table S6. Data on titration of native αB-Cry by Cu^2+^-ions represented in coordinates {[L]; *r*}.** [L] is the equilibrium concentration of Cu^2+^-ions and *r* is a number of the ligand molecules bound the protein molecule.

| [L] (μM) | *r* |
| --- | --- |
| 0.68 | 0.24 |
| 1.18 | 0.51 |
| 3.30 | 0.89 |
| 3.41 | 0.88 |
| 11.6 | 1.12 |
| 11.9 | 1.08 |
| 20.1 | 1.31 |
| 20.4 | 1.28 |
| 29.4 | 1.42 |
| 29.5 | 1.40 |
| 38.5 | 1.54 |
| 38.7 | 1.51 |
| 48.4 | 1.55 |
| 57.9 | 1.61 |
| 58 | 1.60 |
| 67.6 | 1.65 |
| 67.8 | 1.63 |
| 77.4 | 1.68 |
| 77.5 | 1.67 |
| 87.1 | 1.72 |
| 87.3 | 1.70 |
| 107 | 1.73 |
| 127 | 1.78 |
| 147 | 1.80 |
| 167 | 1.79 |
| 186 | 1.85 |
| 236 | 1.87 |
| 286 | 1.92 |

**Table S7. Titration of peroxynitrite modified αB-Cry by Cu^2+^-ions.** The dependence of fluorescence intensity of peroxynitrite modified αB-Cry on the concentration of Cu^2+^-ions.

| Cu^2+^ (μM) | Intensity at 337 nm (a.u.) |
| --- | --- |
| 0 | 170.3 |
| 2.5 | 159.2 |
| 5 | 156.2 |
| 5 | 159.5 |
| 10 | 148.1 |
| 10 | 151.8 |
| 20 | 146.7 |
| 20 | 143.9 |
| 30 | 137.7 |
| 30 | 137.1 |
| 40 | 131.8 |
| 40 | 135.0 |
| 50 | 131.4 |
| 50 | 132.5 |
| 60 | 132.3 |
| 70 | 130.2 |
| 70 | 125.3 |
| 80 | 126.0 |
| 80 | 124.3 |
| 90 | 123.1 |
| 90 | 122.4 |
| 100 | 124.3 |
| 100 | 121.8 |
| 120 | 122.4 |
| 140 | 121.9 |
| 160 | 120.4 |
| 180 | 119.8 |
| 200 | 117.9 |
| 250 | 116.2 |
| 300 | 115.7 |

**Table S8. Data on titration of peroxynitrite modified αB-Cry by Cu^2+^-ions represented in coordinates {[L]; *r*}.** [L] is the equilibrium concentration of Cu^2+^-ions and *r* is a number of the ligand molecules bound the protein molecule.

| [L] (μM) | *r* |
| --- | --- |
| 1.55 | 0.460 |
| 2.37 | 0.351 |
| 4.57 | 0.725 |
| 5.47 | 0.604 |
| 13.6 | 0.859 |
| 14.2 | 0.768 |
| 21.9 | 1.083 |
| 22.0 | 1.062 |
| 30.6 | 1.255 |
| 31.4 | 1.152 |
| 40.5 | 1.267 |
| 40.8 | 1.233 |
| 50.7 | 1.239 |
| 59.0 | 1.466 |
| 60.2 | 1.307 |
| 68.7 | 1.501 |
| 69.2 | 1.444 |
| 78.3 | 1.560 |
| 78.5 | 1.539 |
| 88.1 | 1.582 |
| 88.7 | 1.500 |
| 108 | 1.560 |
| 128 | 1.578 |
| 148 | 1.628 |
| 168 | 1.645 |
| 187 | 1.707 |
| 237 | 1.764 |
| 287 | 1.781 |
